# Nanoparticle albumin-bound paclitaxel and ramucirumab versus paclitaxel and ramucirumab as second-line chemotherapy for unresectable advanced or recurrent gastric cancer: a multicenter, propensity score-matched analysis (CROSS SELL study)

**DOI:** 10.1007/s10147-022-02114-y

**Published:** 2022-01-28

**Authors:** Akio Nakasya, Yuya Hagiwara, Tatsuki Ikoma, Yusuke Kurioka, Toshihiko Matsumoto, Yoshiyuki Yamamoto, Takao Tsuduki, Takeshi Kajiwara, Toshikazu Moriwaki, Tomohiro Nishina, Natsumi Yamashita, Ichinosuke Hyodo

**Affiliations:** 1 Department of Gastrointestinal Medical Oncology, National Hospital Organization Shikoku Cancer Center, 160 Kou, Minamiumemoto-machi, Matsuyama, Ehime 791-0280 Japan; 2 Department of Gastroenterology, Faculty of Medicine, University of Tsukuba, Tsukuba, Ibaraki Japan; 3 Department of Medical Oncology, Kobe City Medical Center General Hospital, Kobe, Hyogo Japan; 4 Department of Internal Medicine, Himeji Red Cross Hospital, Himeji, Hyogo Japan; 5 Section of Cancer Prevention and Epidemiology, Clinical Research Center, National Hospital Organization Shikoku Cancer Center, Matsuyama, Ehime Japan

**Keywords:** Gastric cancer, Chemotherapy, Albumin-bound paclitaxel, Paclitaxel, Ramucirumab, Propensity score

## Abstract

**Background:**

Paclitaxel plus ramucirumab (PTX + RAM) is the standard second-line chemotherapy for unresectable advanced or recurrent gastric cancer (AGC). Nanoparticle albumin-bound paclitaxel (nab-PTX) is an improved, more convenient form of PTX and is non-inferior to PTX. Although some retrospective and single-arm phase II studies regarding nab-PTX + RAM have been reported, comparative studies are lacking. Here, we compared the efficacy and toxicity of nab-PTX + RAM and PTX + RAM using propensity score matching.

**Methods:**

Clinical data of 265 patients treated for AGC with nab-PTX + RAM or PTX + RAM were retrospectively collected. Nab-PTX was administered at dosages of 100 mg/m^2^, replacing PTX in the standard PTX + RAM regimen. Progression-free survival (PFS), overall survival (OS), and toxicity were compared using 1:1 propensity score matching.

**Results:**

In total, 190 (72%) patients were matched. The median PFS was 5.3 [95% confidence interval (CI) 4.4–6.3] and 4.7 (95% CI 3.2–5.3) months in the nab-PTX + RAM and PTX + RAM groups, respectively [hazard ratio (HR) = 0.76, 95% CI 0.56–1.03, *p* = 0.07]. The median OS was 11.5 (95% CI 9.2–15.0) and 9.9 (95% CI 8.0–12.7) months, respectively (HR = 0.78, 95% CI 0.56–1.07, *p* = 0.12). Grade 3 and 4 neutropenia was observed more frequently in the nab-PTX + RAM group (72% vs. 56%, *p* = 0.03). No treatment-related deaths occurred.

**Conclusions:**

Nab-PTX + RAM exhibited more favorable trends in terms of PFS and OS but was more myelosuppressive than PTX + RAM. As neutropenia is commonly manageable toxicity, nab-PTX + RAM presents a treatment alternative for AGC. Further studies including randomized, controlled studies are warranted.

**Supplementary Information:**

The online version contains supplementary material available at 10.1007/s10147-022-02114-y.

## Introduction

Fluoropyrimidine plus platinum is the recommended standard first-line chemotherapy for unresectable advanced or recurrent gastric cancer (AGC) according to several guidelines [1–3]. The RAINBOW trial [4] demonstrated the superiority of weekly administration of paclitaxel plus ramucirumab (PTX + RAM) over weekly PTX in overall survival (OS) in previously treated AGC patients, after which PTX + RAM became the standard second-line chemotherapy.

Nanoparticle albumin-bound paclitaxel (nab-PTX) is a solvent-free albumin-bound, 130 nm particle form of PTX. Since nab-PTX is free of polyethoxylated castor oil and hydrated ethanol, patients are at lower risk of hypersensitivity compared to when PTX is administered [5]. Therefore, nab-PTX is administered for a shorter time than PTX without premedication and patients with alcohol intolerance can be treated with it [5]. Thus, nab-PTX is more beneficial and convenient than PTX in clinical practice.

The ABSOLUTE trial demonstrated the non-inferiority of weekly nab-PTX to weekly PTX in OS (median, 11.1 vs. 10.9 months; hazard ratio [HR = 0.97, 97.5% confidence interval (CI) 0.76–1.23; non-inferiority *p* = 0.0085], and improved trends in progression-free survival (PFS) (respective median, 5.3 vs. 3.8 months; HR = 0.88, 95% CI 0.73–1.06; *p* = 0.17) and overall response rate (ORR) (33% vs. 24%, *p* = 0.10) as second-line chemotherapy for AGC [5]. In addition, a single-arm phase II trial of nab-PTX + RAM and two retrospective studies of nab-PTX + RAM and PTX + RAM demonstrated promising and similar efficacy, respectively, as second-line chemotherapy for AGC [6–8]. Thus, nab-PTX + RAM is expected to be an alternative treatment to PTX-RAM and could be used instead of PTX + RAM in Japan. However, no comparative study of nab-PTX-RAM and PTX-RAM has been conducted thus far.

Here, we retrospectively analyzed the outcomes of the two treatments using propensity score matching to minimize the bias of patient backgrounds.

## Patients and methods

### Study design and patients

This was a multicenter retrospective study conducted at four institutions (National Hospital Organization, Shikoku Cancer Center, Matsuyama, Ehime, Japan; University of Tsukuba, Tsukuba, Ibaraki, Japan; Himeji Red Cross Hospital, Himeji, Hyogo, Japan; Kobe City Medical Center General Hospital, Kobe, Hyogo, Japan).

The major inclusion criteria were as follows: (a) unresectable advanced or recurrent gastric cancer (including esophagogastric junction cancer), (b) histologically confirmed adenocarcinoma, (c) age: ≥ 20 years, (d) Eastern Cooperative Oncology Group performance status (ECOG PS) of 0–2, (e) evaluable lesions, (f) refractoriness to first-line chemotherapy with the fluoropyrimidine-based regimen (including relapse ≤ 24 weeks after the final administration of fluoropyrimidine-based adjuvant chemotherapy), (g) receiving nab-PTX + RAM or PTX + RAM as second-line chemotherapy, and (h) initiation of second-line chemotherapy between January 2017 and June 2020. The major exclusion criteria were as follows: (i) history of previous administration of taxane or angiogenesis inhibitors and (j) lost to follow-up within 1 month of starting nab-PTX + RAM or PTX + RAM treatment.

### Treatment

The nab-PTX + RAM regimen consisted of administration of 100 mg/m^2^ of nab-PTX intravenously over 30 min on days 1, 8, and 15 along with 8 mg/kg of RAM intravenously on days 1 and 15 of each 28-day cycle. The only premedication permitted was the histamine H1-receptor blocker prior to RAM infusion on days 1 and 15. The PTX + RAM regimen consisted of administration of 80 mg/m^2^ of PTX intravenously over 60 min on days 1, 8, and 15, along with 8 mg/kg of RAM intravenously on days 1 and 15 of each 28-day cycle. The permitted premedication was steroids and histamine H1 and H2-receptor blockers on days 1, 8, and 15. The attending physician determined each patient’s regimen. Nab-PTX + RAM was preferentially selected for the patients with alcohol intolerance, allergies, and underlying conditions (diabetes mellitus, non-tuberculosis mycobacterial infection, and so on) to avoid steroid use. In addition, drug cost, physician’s experience, and institution policy to reduce treatment time affected the choice of regimen. Dose reductions, including the initial dose, and skipping or delaying administration, were also determined according to each physician’s discretion. Treatment was continued until disease progression, unacceptable toxicity, patient refusal, or a physician’s decision to discontinue.

### Endpoints and assessment

Efficacy was evaluated based on PFS, OS, and tumor response. Toxicity was evaluated according to the proportion of patients with Grade 3 or 4 adverse events (AEs). PFS was defined as the time from the initiation of study treatment to disease progression or death due to any cause. Disease progression was defined as radiological or clinical cancer progression. The patients underwent radiological examination every 8 ± 2 weeks. Patients who continued study treatment and discontinued treatment without disease progression were censored at the last confirmation of non-progressive disease by radiological examination. OS was defined as the time from the initiation of study treatment to death due to any cause. Survivors were censored at last contact. Tumor response was assessed based on the Response Evaluation Criteria in Solid Tumors version 1.1 (RECIST ver. 1.1) [9] for patients with measurable lesions. ORR was defined as the proportion of patients who had the best response of complete response or partial response. Disease control rate (DCR) was defined as the proportion of patients who had the best response of complete response, partial response, or stable disease. AEs were graded based on the Common Terminology Criteria for Adverse Events version 5.0 (CTCAE ver. 5.0) [10]. Disease progression was decided by each physician. Tumor response and AEs were also assessed by each physician. Relative dose intensity (RDI) was defined as the ratio of actually delivered dose to the standard dose of drugs from the first to the last administration.

### Statistical analysis

At first, we defined all patients who met the inclusion and exclusion criteria as the original cohort. Patient backgrounds and treatments are both known to affect efficacy and survival [11, 12]. Therefore, we used 1:1 propensity score matching to balance the patient background characteristics between the two treatment groups. Propensity scores were estimated using a multivariable logistic regression model that included six covariates [ECOG PS, histological tumor differentiation, presence of primary tumor, number of metastatic sites, peritoneal metastasis, and serum lactate dehydrogenase (LDH) level]. These covariates were identified by the multivariate analysis for OS in the original cohort (cutoff *p* < 0.20), and adopted confirming correspondence to the reported prognostic factors [11–13]. Then, the patients matched using the scores were defined as the matched cohort. The patient characteristics of the two treatment groups were compared using standardized differences. The efficacy and toxicity of the groups were compared in the matched cohort. Inverse probability of treatment weighting (IPTW) analysis was also performed as sensitivity analysis in the original cohort. Survival curves were generated using the Kaplan–Meier method. The PFS and OS rates were compared using the log-rank test. HR and 95% CI were estimated using the Cox proportional hazards model, as was subgroup univariate analysis. Fisher’s exact test was used to compare the ORR, DCR and toxicity of the groups. Wilcoxon rank sum test was used to compare the RDI and actually delivered dose of the groups. The follow-up time was estimated using the reverse Kaplan–Meier method. A standardized difference of < 0.10 was defined as statistically not different or well balanced [14]. All *p* values were two-sided, and statistical significance was set at *p* < 0.05. Statistical analyses were performed using JMP^®^ 13 (SAS Institute Inc., Cary, NC, USA) and SAS software (version 9.4; SAS Institute Inc., Cary, NC, USA).

## Results

### Patients

**Fig. 1:**
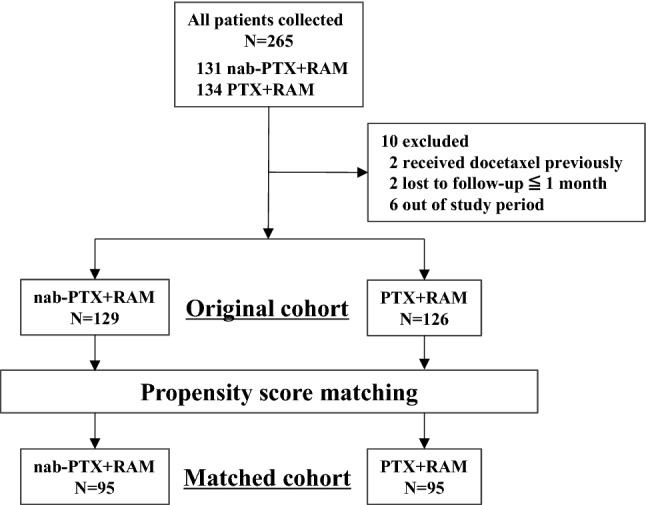
Consolidated Standards of Reporting Trial (CONSORT) flow diagram. *Nab-PTX* + *RAM* nanoparticle albumin-bound paclitaxel plus ramucirumab, *PTX* + *RAM* paclitaxel plus ramucirumab, *N* number of patients

The flow chart for patient selection is shown in Fig. 1. Data of 265 patients treated with nab-PTX + RAM or PTX + RAM as second-line chemotherapy were collected from four institutions. After ineligible patients were excluded, 129 and 126 patients from the nab-PTX + RAM and PTX + RAM groups, respectively, were analyzed as the original cohort. The background characteristics of the patients in each treatment group are shown in Table 1. There were some imbalances between the two treatment groups, with the nab-PTX + RAM group exhibiting worse background characteristics (undifferentiated tumor, multiple number of metastatic sites, presence of primary tumor, peritoneal metastasis, and a high LDH level). After 1:1 propensity score matching, 190 (72%) patients were matched as 95 pairs. In this matched cohort, the patient background characteristics were balanced between the two treatment groups (Table 1).

**Table 1:** Patient background characteristics

	Original cohort		Matched cohort		Original cohort	Matched cohort
	nab-PTX + RAM	PTX + RAM	nab-PTX + RAM	PTX + RAM	Standardized difference	
	*N* = 129	*N* = 126	*N* = 95	*N* = 95		
Age						
Median (range)	67 (31–90)	69 (36–85)	67 (41–90)	69 (36–85)		
< 65	55	41	38	30	0.21	0.17
≧ 65	74	85	57	65		
Sex						
Male	94	92	69	69	0	0
Female	35	34	26	26		
ECOG PS						
0	57	45	40	36	0.17	0.08
1	66	68	52	51	0.06	0.02
2	6	13	3	8	0.22	0.22
Tumor differentiation						
Differentiated	42	52	36	36	0.18	0
Undifferentiated	87	74	59	59		
Presence of primary tumor						
No	46	64	37	40	0.31	0.06
Yes	83	62	58	55		
Number of metastatic sites						
0–1	48	55	40	38	0.13	0.04
2 ≦	81	71	55	57		
Liver metastasis						
No	91	93	63	69	0.05	0.13
Yes	38	34	32	26		
Peritoneal metastasis						
No	48	55	41	41	0.13	0
Yes	81	71	54	54		
Massive ascites						
No	116	116	86	89	0.07	0.11
Yes	13	10	9	6		
Measurable lesions						
No	57	69	42	47	0.21	0.10
Yes	72	57	53	48		
HER2 status						
Negative (unknown)	107 (1)	104 (1)	77	77 (1)	0.01	0.02
Positive	21	21	18	17		
High AST level						
No	88	98	67	73	0.22	0.14
Yes	41	28	28	22		
High ALP level						
No (unknown)	74 (1)	87	56 (1)	64	0.23	0.15
Yes	54	39	38	31		
High LDH level						
No	67	83	59	54	0.29	0.10
Yes	62	43	36	41		
Low albumin level						
No	35	26	25	19	0.15	0.15
Yes	94	100	70	76		
Low sodium level						
No	117	114	86	88	0.01	0.07
Yes	12	12	9	7		
High neutrophil level						
No (unknown)	100	98 (1)	74	75 (1)	0.03	0.05
Yes	29	27	21	19		
Low lymphocyte level						
No (unknown)	105	98 (1)	79	73 (1)	0.07	0.13
Yes	24	27	16	21		
First-line chemotherapy						
Fluoropyrimidine	129	126	95	95	0	0
Platinum	115	98	84	75	0.31	0.25

*Nab-PTX* + *RAM* nanoparticle albumin-bound paclitaxel plus ramucirumab, *PTX* + *RAM* paclitaxel plus ramucirumab, *N* number of patients, *ECOG PS* Eastern Cooperative Oncology Group performance status, *HER2* human epidermal growth factor receptor 2, *AST* aspartate aminotransferase, *ALP* alkaline phosphatase, *LDH* lactate dehydrogenase, massive ascites means that ascites exist from the surface of the liver to the pelvic cavity continuously

### Efficacy

In the matched cohort, 94 (99%) and 95 (100%) patients in the nab-PTX-RAM and PTX + RAM groups, respectively, discontinued study treatment. The most common reason for treatment discontinuation was disease progression, which affected 88 (93%) patients in each group (Online Resource 1). At the data cutoff time for analyses (September 2021), the median follow-up time for OS was 24.8 and 29.9 months in the nab-PTX + RAM and PTX + RAM groups, respectively. During the study period, 72 (76%) and 82 (86%) patients died in the nab-PTX + RAM and PTX + RAM groups, respectively.

**Fig. 2:**
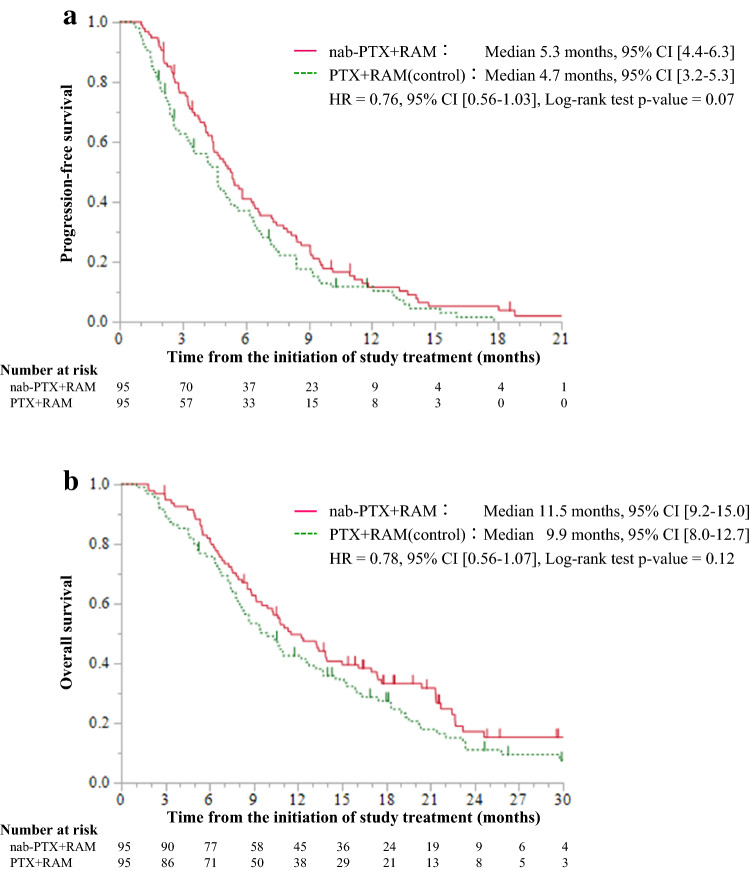
Kaplan–Meier curves of progression-free survival (**a)** and overall survival (**b)** in propensity score-matched patients. *Nab-PTX* + *RAM* nanoparticle albumin-bound paclitaxel plus ramucirumab, *PTX* + *RAM* paclitaxel plus ramucirumab, *HR* hazard ratio, *CI* confidence interval

**Fig. 3:**
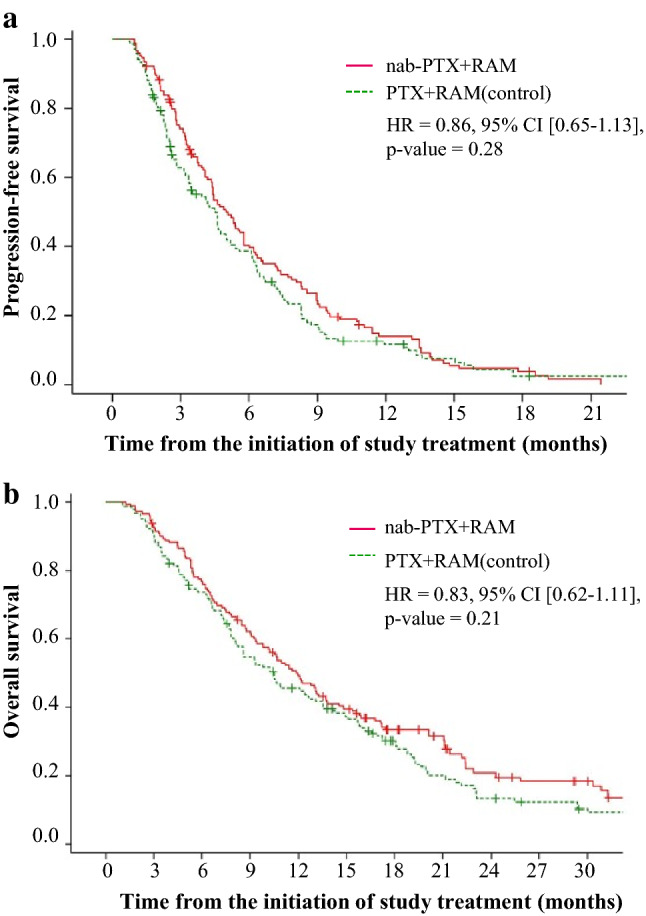
Kaplan–Meier curves of progression-free survival (**a**) and overall survival (**b)** in Inverse Probability of Treatment Weighting (IPTW) patients. *Nab-PTX* + *RAM* nanoparticle albumin-bound paclitaxel plus ramucirumab, *PTX* + *RAM* paclitaxel plus ramucirumab, *HR* hazard ratio, *CI* confidence interval

**Fig. 4:**
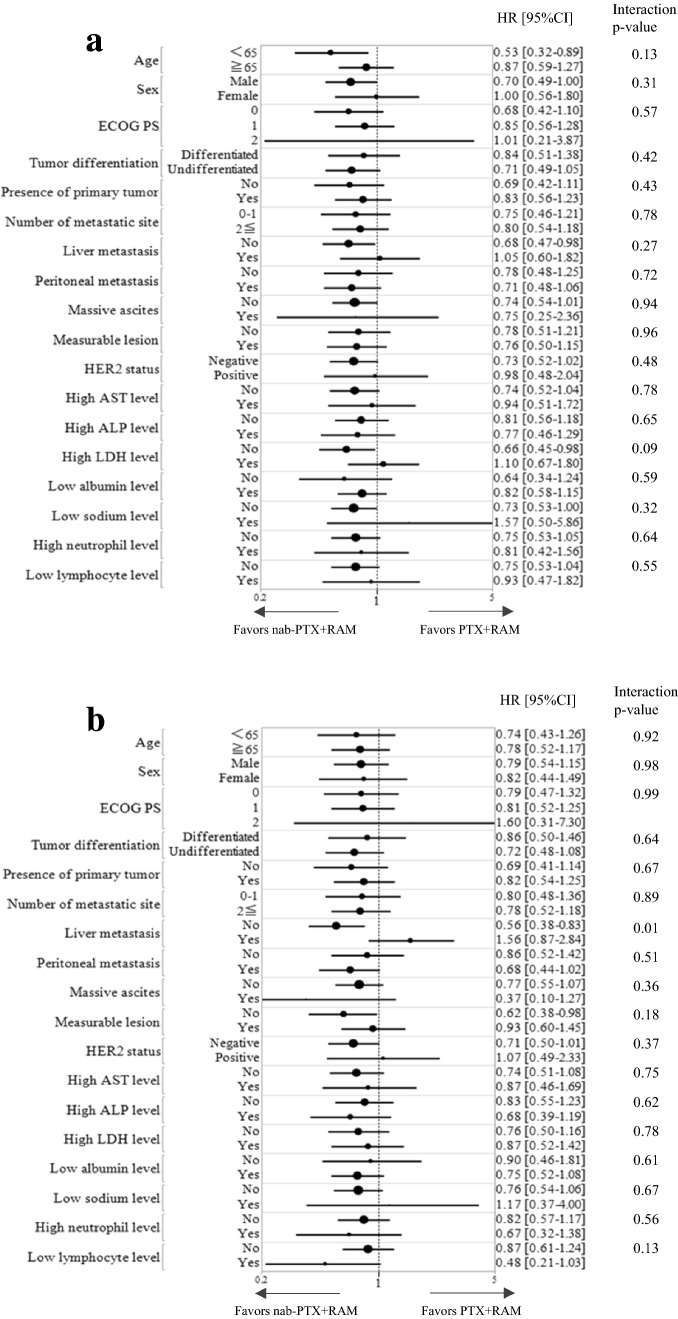
Forest plots for subgroup univariate analyses of progression-free survival (**a)** and overall survival (**b)**. The size of the center circle is proportional to the number of patients in the subgroup. *Nab-PTX* + *RAM* nanoparticle albumin-bound paclitaxel plus ramucirumab, *PTX* + *RAM* paclitaxel plus ramucirumab, *HR* hazard ratio, *CI* confidence interval, *ECOG PS* Eastern Cooperative Oncology Group performance status, *HER2* human epidermal growth factor receptor 2, *AST* aspartate aminotransferase, *ALP* alkaline phosphatase, *LDH* lactate dehydrogenase

The median PFS was 5.3 (95% CI 4.4–6.3) and 4.7 (95% CI 3.2–5.3) months in the nab-PTX + RAM and PTX + RAM groups, respectively (HR = 0.76, 95% CI 0.56–1.03, *p* = 0.07) (Fig. 2a). The median OS was 11.5 (95% CI: 9.2–15.0) months in the nab-PTX + RAM group and 9.9 (95% CI 8.0–12.7) months in the PTX + RAM group (HR = 0.78, 95% CI 0.56–1.07, *p* = 0.12) (Fig. 2b). According to IPTW analysis, the HR of nab-PTX + RAM versus PTX + RAM for PFS was 0.86 (95% CI 0.65–1.13, *p* = 0.28) and that for OS was 0.83 (95% CI 0.62–1.11, *p* = 0.21) (Fig. 3). Subgroup analyses generally tended to favor nab-PTX + RAM in terms of PFS and OS (Fig. 4). The ORR was 40% and 37% in the nab-PTX + RAM and PTX + RAM groups, respectively (*p* = 0.84), and the DCR was 87% and 77% (*p* = 0.29), respectively (Table 2). Online Resource 2 shows the waterfall plots of tumor shrinkage of each patient. The mean depth of response was −21% (95% CI −12% to −30%) in the nab-PTX + RAM group and −14% (95% CI −3% to −25%) in the PTX + RAM group.

**Table 2:** Tumor response

	nab-PTX + RAM		PTX + RAM		Fisher’s exact test
	*N* = 53	%	*N* = 48	%	*p* value
Best overall respose					
Complete response	0	0	1	2	
Partial response	21	40	17	35	
Stable disease	25	47	19	40	
Progressive disease	6	11	11	23	
Not evaluated	1	2	0	0	
Overall response rate	21	40	18	37	0.84
Disease control rate	46	87	37	77	0.29

*Nab-PTX* + *RAM* nanoparticle albumin-bound paclitaxel plus ramucirumab, *PTX* + *RAM* paclitaxel plus ramucirumab, *N* number of patients

### Toxicity

The proportion of patients with Grade 3 or 4 AEs in the nab-PTX + RAM group was higher than in the PTX + RAM group (86% vs. 69%, *p* < 0.01) (Table 3). The most common AE was a decrease in neutrophil count and more patients in the nab-PTX + RAM group developed this condition than in the PTX + RAM group (72% vs. 56%, *p* = 0.03). The incidence of febrile neutropenia was comparable between the groups (8% vs. 9%, *p* = 1.00). Grade 3 and 4 anemia tended to occur more frequently in the nab-PTX + RAM group than in the PTX + RAM group (26% vs. 17%, *p* = 0.15). No hypersensitivity reactions or treatment-related deaths occurred during either treatment.

**Table 3:** Adverse events ≥ Grade3

	nab-PTX + RAM		PTX + RAM		Fisher’s exact test
	*N* = 95	%	*N* = 95	%	*p* value
Any adverse events	82	86	66	69	< 0.01
Hematological					
Neutrophil count decreased	68	72	53	56	0.03
White blood cell decreased	30	32	30	32	1.00
Anemia	25	26	16	17	0.15
Platelet count decreased	5	5	4	4	1.00
Non-hematological					
Febrile neutropenia	8	8	9	9	1.00
Peripheral sensory neuropathy	4	4	9	9	0.24
Fatigue	4	4	3	3	1.00
Anorexia	3	3	2	2	1.00
Mucositis oral	1	1	1	1	1.00
Diarrhea	1	1	1	1	1.00
Edema limbs	1	1	2	2	1.00
Nausea	0	0	1	1	1.00
Vomiting	0	0	1	1	1.00
Bone infection	1	1	0	0	1.00
Pneumonitis	0	0	1	1	1.00
Special interest					
Hypertension	11	12	7	7	0.45
Proteinuria	4	4	5	5	1.00
Gastrointestinal hemorrhage	4	4	0	0	0.12
Thromboembolic event	2	2	2	2	1.00
Gastrointestinal perforation	0	0	2	2	0.49

*Nab-PTX* + *RAM* nanoparticle albumin-bound paclitaxel plus ramucirumab, *PTX* + *RAM* paclitaxel plus ramucirumab, *N* number of patient

### RDI and actually delivered dose

The median RDI of taxane was 63% [interquartile range (IQR) 49–83] and 66% (IQR 51–83) in the nab-PTX + RAM and PTX + RAM groups, respectively (*p* = 0.50). The median actually delivered dose of taxane was 190 mg/m^2^/cycle (IQR 147–249) and 158 mg/m^2^/cycle (IQR 121–199) in the nab-PTX + RAM and PTX + RAM groups, respectively (*p* < 0.01). The median RDI of RAM was 85% (IQR 67–100) and 89% (IQR 71–100) in the nab-PTX + RAM and PTX + RAM groups, respectively (*p* = 0.28). The median actually delivered dose of RAM was 169 mg/m^2^/cycle (IQR: 134–200) and 178 mg/m^2^/cycle (IQR: 141–200) in the nab-PTX + RAM and PTX + RAM groups, respectively (*p* = 0.28).

### Post-study treatment

Approximately 81% and 64% of patients in the nab-PTX + RAM and PTX + RAM groups received third-line chemotherapy and 40% and 34% received fourth-line chemotherapy, respectively. The proportions of patients by treatments in the nab-PTX + RAM and PTX + RAM groups were as follows: immune checkpoint inhibitors (nivolumab or pembrolizumab), 72% and 57%; irinotecan, 29% and 27%; trifluridine/tipiracil, 24% and 11%; and trastuzumab deruxtecan, 11% and 5% (Online Resource 3).

## Discussion

To our knowledge, this is the first study to compare the efficacy and toxicity of nab-PTX + RAM versus PTX + RAM as second-line chemotherapy in multicenter and propensity score-matched patients with AGC. Nab-PTX + RAM exhibited more favorable trends in efficacy and more myelosuppressive AEs in toxicity, compared with PTX + RAM.

In terms of efficacy, nab-PTX + RAM exhibited favorable trends in PFS and OS compared to PTX + RAM, despite no statistically significant differences being observed. Sensitivity analyses using IPTW revealed similar results. The PFS and OS rates of the PTX + RAM group in our study were comparable to those observed in the RAINBOW trial [4]. In addition, as shown in Online Resource 4, the PFS and OS of nab-PTX + RAM in the previous two retrospective studies appeared favorable compared with those of PTX + RAM, or at least non-inferior. Forest plots of subgroup analyses of PFS and OS generally tended to favor nab-PTX + RAM. Nab-PTX appeared to have a better effect on peritoneal metastasis than PTX according to the exploratory subgroup analysis of the ABSOLUTE trial [15] and a retrospective study on nab-PTX + RAM and PTX-RAM [7], presumably due to the drug formulation of nab-PTX. However, this interaction between peritoneal metastasis and treatment was not observed in our study or in another retrospective study [8], and remained controversial. The ongoing P-SELECT trial (WJOG10617G) [16], a multicenter randomized phase II trial of nab-PTX + RAM versus PTX + RAM in second-line chemotherapy for AGC patients with peritoneal metastasis, may elucidate this finding. The current study demonstrated that ORR, DCR, and depth of response were comparable between the two treatments, which is equivalent to the results of other studies [7, 8].

Regarding toxicity, the proportion of patients with Grade 3 and 4 AEs was statistically higher in the nab-PTX + RAM group than in the PTX + RAM group. These were predominantly manageable hematological toxicities, such as a neutrophil count decrease and anemia. Although the RDIs of taxane and RAM were compatible between the two treatments, the actually delivered dose of taxane was statistically higher in the nab-PTX + RAM group than that in the PTX + RAM group, which seemed to explain the increase of hematological toxicities in the nab-PTX + RAM group. A retrospective study showed that the neutrophil count decrease observed in the patients receiving weekly PTX was strongly associated with better efficacy [17], and a prospective study demonstrated that PFS rates were better in patients treated with neutropenia-guided dose-escalation weekly PTX than in those receiving standard-dose weekly PTX [18]. Taken together with these findings, the higher incidence of neutrophil count decreased in nab-PTX + RAM might not be a disadvantage for efficacy. Considering that less than 10% of patients discontinued study treatment due to unacceptable toxicity and that treatment-related death did not occur due to either treatment, both treatments appear feasible in clinical practice. No hypersensitivity reactions were observed in either treatment group. As indicated in “Methods”, the only permitted premedication for nab-PTX + RAM was a histamine H1-receptor blocker, making this result remarkable. In addition, nab-PTX can be used in patients with alcohol intolerance and has a shorter administration time. Nab-PTX + RAM is, therefore, more convenient than PTX + RAM in clinical practice.

The proportions of patients who received later-line chemotherapy, primarily immune checkpoint inhibitors and trifluridine/tipiracil, differed between the two treatment groups. We speculated the main reason for the difference was the approval of nivolumab and trifluridine/tipiracil during the study period. They were approved as treatment for AGC in Japan in September 2017 and August 2019, respectively. As nab-PTX + RAM has been currently selected more frequently, the patients administered nab-PTX + RAM were considered to have more chance to access these drugs.

The present study has several limitations. First, this was a non-randomized retrospective study, and the sample size was small. Although we used propensity score matching to balance the patient background characteristics in the treatment groups, we could not adjust for unmeasured confounding factors, which might have affected the results. In addition, the sample size shrank further when matched pairs were made, which weakened the statistical power. Second, as mentioned above, the difference in proportion receiving post-study treatment might have partly affected OS. Considering this bias, OS data should be interpreted with caution.

In conclusion, nab-PTX + RAM exhibited favorable trends in terms of PFS and OS compared with PTX + RAM. Although hematological toxicity was of concern, it was manageable. Based on these results, further studies including randomized-controlled studies are warranted.

## Supplementary Information

Below is the link to the electronic supplementary material.

Supplementary file2 (PPTX 815 kb)

Supplementary file1 (DOCX 29 kb)

Supplementary file3 (DOCX 28 kb)

Supplementary file4 (DOCX 35 kb)

## Data Availability

The datasets analyzed during the current study are available from the corresponding author on reasonable request.
