# Supplementary material for: Nanoparticle albumin-bound paclitaxel and ramucirumab versus paclitaxel and ramucirumab as second-line chemotherapy for unresectable advanced or recurrent gastric cancer: a multicenter, propensity score-matched analysis (CROSS SELL study)
Source: Int J Clin Oncol. 2022 Jan 28;27(4):684–94. doi: 10.1007/s10147-022-02114-y (PMC8956527; doi:10.1007/s10147-022-02114-y)
Supplement: Supplementary file 1 — Supplementary file2 (PPTX 815 kb) [file 10147_2022_2114_MOESM1_ESM.pptx]

## Slide 1
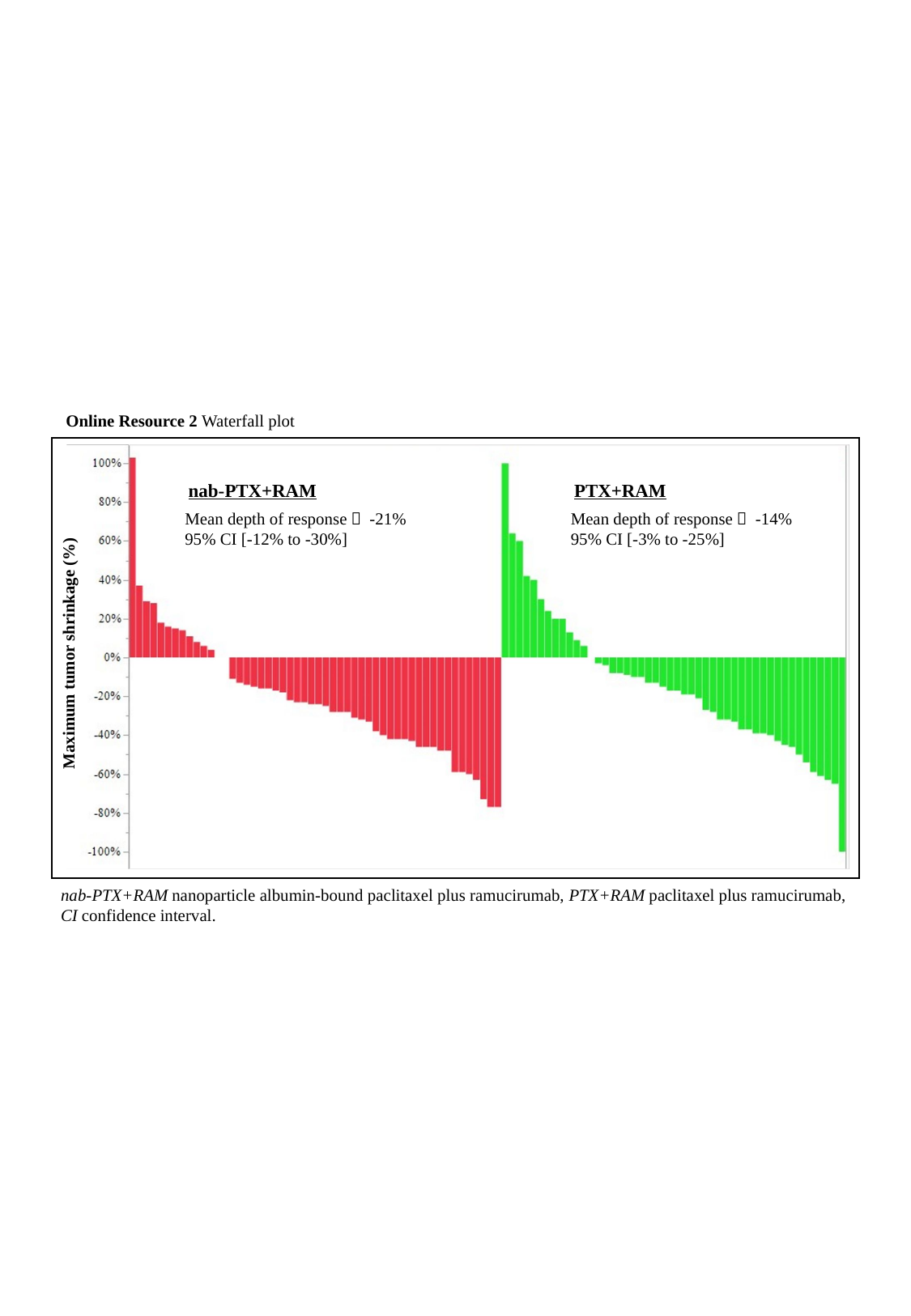

Online Resource 2 Waterfall plot
nab-PTX+RAM
PTX+RAM
Mean depth of response： -21%
95% CI [-12% to -30%]
Mean depth of response： -14%
95% CI [-3% to -25%]
Maximum tumor shrinkage (%)
nab-PTX+RAM nanoparticle albumin-bound paclitaxel plus ramucirumab, PTX+RAM paclitaxel plus ramucirumab,
CI confidence interval.
