# Supplementary material for: Nanoparticle albumin-bound paclitaxel and ramucirumab versus paclitaxel and ramucirumab as second-line chemotherapy for unresectable advanced or recurrent gastric cancer: a multicenter, propensity score-matched analysis (CROSS SELL study)
Source: Int J Clin Oncol. 2022 Jan 28;27(4):684–94. doi: 10.1007/s10147-022-02114-y (PMC8956527; doi:10.1007/s10147-022-02114-y)
Supplement: Supplementary file 2 [file 10147_2022_2114_MOESM2_ESM.docx]

Online Resource 3 Post-study treatment

	nab-PTX+RAM		PTX+RAM
	N=95	%	N=95
Third-line chemotherapy	77	81	61
Fourth-line chemotherapy	38	40	32
Nivolumab or Pembrolizumab	68	72	54
Irinotecan	28	29	26
Trifluridine/tipiracil	23	24	10
Trastuzumab deruxtecan	10	11	5
Others	8	8	8

Nab-PTX+RAM nanoparticle albumin-bound paclitaxel plus ramucirumab, *PTX+RAM* paclitax
