# Supplementary material for: Nanoparticle albumin-bound paclitaxel and ramucirumab versus paclitaxel and ramucirumab as second-line chemotherapy for unresectable advanced or recurrent gastric cancer: a multicenter, propensity score-matched analysis (CROSS SELL study)
Source: Int J Clin Oncol. 2022 Jan 28;27(4):684–94. doi: 10.1007/s10147-022-02114-y (PMC8956527; doi:10.1007/s10147-022-02114-y)
Supplement: Supplementary file 3 [file 10147_2022_2114_MOESM3_ESM.docx]

Online Resource 4 Comparison of efficacy from previous studies

Trial or study	nab-PTX+RAM				PTX+	
	N	Median PFS months (95%CI)	Median OS months (95%CI)	ORR %	N	Median PFS months (95%CI)
RAINBOW trial (Ref.4)					330	4.4 (4.2-5.3)
Single-arm phase II trial (Ref.6)	42	7.6 (5.4-8.1)	not reached	55		
Retrospective study 2019 (Ref.7)	35	4.6 (3.5-8.0)	11.4 (7.4-15.2)	38	93	4.1 (3.3-4.6)
Retrospective study 2020 (Ref.8)	113	3.9 (3.4-4.3)	10.9 (9.3-12.7)	34	138	3.9 (3.1-4.7)
Our study	95	5.3 (4.4-6.3)	11.5 (9.2-15.0)	40	95	4.7 (3.2-5.3)

Nab-PTX+RAM nanoparticle albumin-bound paclitaxel plus ramucirumab, *PTX+RAM* pac[illegible] ramucirumab, *N* number of patients, *PFS* progression-free survival, *OS* overall survival, *O*[illegible]
[illegible]
