# Supplementary material for: Nanoparticle albumin-bound paclitaxel and ramucirumab versus paclitaxel and ramucirumab as second-line chemotherapy for unresectable advanced or recurrent gastric cancer: a multicenter, propensity score-matched analysis (CROSS SELL study)
Source: Int J Clin Oncol. 2022 Jan 28;27(4):684–94. doi: 10.1007/s10147-022-02114-y (PMC8956527; doi:10.1007/s10147-022-02114-y)
Supplement: Supplementary file 4 [file 10147_2022_2114_MOESM4_ESM.docx]

Online Resource 1 Reasons for discontinuation of study treatment

	nab-PTX+RAM		PTX+RAM
	N=95	%	N=95
Continuations	1	1	0
Discontinuations	94	99	95
Disease progression	88	93	88
Radiological progression	76	80	78
Clinical progression	10	11	7
Death due to disease progression	2	2	3
Without disease progression	6	6	7
Unacceptable toxicity	5	5	6
Physician's decision	1	1	1

(Unacceptable toxicity)

nab-PTX+RAM: 1 febrile neutropenia, 1 platelet count decreased, 1 peripheral sensory neuropathy, 1 bone infection, 1 gastrointestinal hemorrhage

PTX+RAM: 1 febrile neutropenia, 1 fatigue, 3 peripheral sensory neuropathy, 1 pneumonitis

(Physician’s decision)

nab-PTX+RAM: 1 conversion surgery

PTX+RAM: 1 complete response

Nab-PTX+RAM nanoparticle albumin-bound paclitaxel plus ramucirumab, *PTX+RAM* paclitax [illegible]
